# Sensorimotor abilities predict on-field performance in professional baseball

**DOI:** 10.1038/s41598-017-18565-7

**Published:** 2018-01-08

**Authors:** Kyle Burris, Kelly Vittetoe, Benjamin Ramger, Sunith Suresh, Surya T. Tokdar, Jerome P. Reiter, L. Gregory Appelbaum

**Affiliations:** 1 Department of Statistical Science, Duke University, Durham, NC 27708 USA; 2 Department of Psychiatry and Behavioral Science, Duke University School of Medicine, Durham, 27710 NC USA; 3 Urbaniak Sports Sciences Institute, Duke University, Durham, NC 27705 United States

## Abstract

Baseball players must be able to see and react in an instant, yet it is hotly debated whether superior performance is associated with superior sensorimotor abilities. In this study, we compare sensorimotor abilities, measured through 8 psychomotor tasks comprising the Nike Sensory Station assessment battery, and game statistics in a sample of 252 professional baseball players to evaluate the links between sensorimotor skills and on-field performance. For this purpose, we develop a series of Bayesian hierarchical latent variable models enabling us to compare statistics across professional baseball leagues. Within this framework, we find that sensorimotor abilities are significant predictors of on-base percentage, walk rate and strikeout rate, accounting for age, position, and league. We find no such relationship for either slugging percentage or fielder-independent pitching. The pattern of results suggests performance contributions from both visual-sensory and visual-motor abilities and indicates that sensorimotor screenings may be useful for player scouting.

## Introduction

Ted Williams, one of the most legendary baseball players of all time, once said, “I think without question the hardest single thing to do in sport is to hit a baseball”. Advances in sport science continue to validate Williams’ claim; hitting a pitched baseball places incredible demands on athletes’ visual systems. We now know that Major League Baseball (MLB) pitches move at speeds near the processing limits of the vestibular-ocular tracking^1^, leaving the batter with mere milliseconds to decipher the pitch, project its trajectory, decide to swing, and coordinate the timing and trajectory of a 2.25-inch diameter bat. The immense difficulty of this task is underscored by the fact that players who hit successfully on less than a third of their at-bats can receive hundred million dollar contracts in today’s free-agent market.

Pitching, while equally demanding, draws upon a fundamentally different skill set. Pitchers attempt to deny batters effective contact with the ball while projecting it through the strike zone 60 feet away. Despite the need to visualize the strike zone, it has been argued that motor demands, such as controlling the speed, spin, and location of the ball are more important for pitching success than visual requirements^2^.

Given the substantial role of visual and motor demands in baseball (henceforth called “sensorimotor skills”), there has been a concerted effort to determine which elements of the perception-action cycle contribute to successful baseball performance^3^. However, the combination of noisy game statistics and costly sample acquisition makes inferring meaningful relationships difficult. Although a small number of studies have reported links between superior baseball statistical production and better visual reaction times^4^, dynamic stereoacuity^5^, binocular divergence^6^, and visual recognition^7,8^, they are based on small sample sizes and/or appear in conference proceedings, rather than peer reviewed articles.

A more common approach for inferring the sensorimotor abilities important for baseball performance involves studying the difference between professionals, amateurs, and non-athletes. This literature, and the larger debate across all sports, centers on the question of whether athletes possess inherently better visual-system physiology (so-called “visual hardware” that allows for the reception of visual information), or if differences are restricted to enhanced perceptual-cognitive abilities that can be shaped through practice (so-called “visual software” involved in processing of visual information)^9^. Some studies have found that expert baseball players possess superior visual acuity^10^, enhanced contrast sensitivity^11^, better peripheral vision^12^, and better visual tracking abilities^13^ than non-athlete controls. While these studies indicate that superior batters possess superior visual system physiology, the preponderance of evidence in the literature concludes that, in the absence of hardware differences, expert performance is subserved by superior abilities to process and act upon visual information. For example, past research found that expert athletes demonstrated more adept anticipation, pattern recognition, and visual search skills than non-experts^14–16^. Nevertheless, given the challenges inherent in doing research with high-level athlete populations, the contribution of hardware and software to expert performance remains an open question.

Between 2011 and 2015, the Nike Sensory Stations were developed and utilized as a tool to quantitatively evaluate athlete visual and motor skills. Participants filled out a registry of information about themselves and completed a battery of nine visual-motor tasks administered under standardized conditions with video instructions and conducted by trained and certified administrators. In addition, participants reported hand and foot preference and completed the Miles test to assess eye-dominance. Data from these assessments were maintained on a central database and used to provide sport-specific normative information to individuals about their relative abilities and to monitor learning when coupled with sensorimotor training interventions.

Past research with the Sensory Stations has demonstrated that the battery of tests is reliable^17,18^ and cross-validated^19^, with some tasks demonstrating linear improvements with practice over multiple sessions^20^. Improved performance on this battery has been seen following sports vision training interventions^21^ and has been linked to baseball batting expertise, with professional baseball hitters showing better performance on measure of visual sensitivity relative to pitchers^22^. Furthermore, reduced performance on these tasks has been associated with an increased likelihood of sustaining head impacts during practices and games for American collegiate football players^23^. In addition, Poltavski and Biberdorff^24^ found that better performance on measures of dynamic visual acuity and visual motor control accounted for nearly 70% of the variability in goals scored over two seasons in a sample of 19 men’s and 19 women’s collegiate hockey players. Collectively, past research reviewed by^25^ suggests that this battery may serve as a useful tool for understanding human performance, warranting further investigation into the sensorimotor characteristics of athletes and their relation to performance outcomes.

## Methods

In the current study, Sensory Station assessments from 252 professional baseball players collected in 2012 and 2013 were compared to game statistics to evaluate the relationship between sensorimotor skills and baseball production. For each player, game statistics from the season after testing were acquired along with information about their league(s) of participation. All data were shared with the research team under a secondary-data protocol approved by the Duke University Institutional Review Board [IRB B0706]. Under this protocol, all data were collected for “real world use,” without informed consent, and shared with the research team after removal of all protected health information (PHI). As such, these data conform to U.S. Department of Health and Human Services, “Regulatory considerations regarding classification of projects involving real world data^26^”.

### Sensorimotor Assessments

The Sensory Stations consist of a battery of nine computerized sensorimotor tasks, each designed to evaluate a specific facet of a participant’s visual-motor abilities. Brief descriptions for each task are provided below, and detailed descriptions can be found in Supplementary Material 1. Behavioral performance distributions on these measures is shown in Supplemental Material 2.

- The **Visual Clarity** task measures visual acuity for fine details at a distance.
- The **Contrast Sensitivity** task measures the minimum resolvable difference in contrast at a distance.
- The **Depth Perception** task measures how quickly and accurately participants are able to detect differences in depth at a distance using liquid crystal glasses.
- The **Near-Far Quickness** task measures the number of near and far targets that can be correctly reported in 30 seconds.
- The **Target Capture** task measures the speed at which participants can shift attention and recognize peripheral targets.
- The **Perception Span** task measures the ability to remember and recreate visual patterns.
- The **Eye Hand Coordination** task measures the speed at which participants can make visually-guided hand responses to rapidly changing targets.
- The **Go/No-Go** task measures the ability to execute and inhibit visually guided hand responses in the presence of “go” and “no-go” stimuli.
- The **Reaction Time** task measures how quickly participants react and respond to a simple visual stimulus.

### Response Variables

The sensorimotor assessments performed by the Nike Sensory Stations serve as our best measurement of a player’s underlying sensorimotor abilities. Similarly, a player’s game statistics are the best indication of his on-field performance. In this study, we use on-base percentage (OBP), walk rate (BB%), strikeout rate (K%), and slugging-percentage (SLG) to measure the performance of batters. In addition, we use fielder-independent pitching (FIP) to measure the performance of pitchers. Below are brief descriptions of each of these statistics and our motivation for using them as response variables in our models. Illustrations of the distribution of these variables are shown in Supplementary Material 3.

**On-Base Percentage** measures a player’s propensity to reach base. On-base percentage is defined as

1 $$OBP=\frac{Hits+Walks+Hit\,By\,Pitch}{At\,Bats+Walks+Hit\,By\,Pitch+Sacrifice\,Flies}$$

Equation 1: Definition of On-Base Percentage.

On-base percentage is a simple and widely used metric for player evaluation, since frequently reaching base gives the offense more opportunities to score runs. Players with high on-base percentages consistently make effective contact with the ball and draw walks. As such, on-base percentage offers a robust measure of player productivity and a useful statistic by which to evaluate the relationship between sensorimotor abilities and on-field performance.

**Walk Rate** measures a player’s propensity to draw walks. Walk rate is defined as

2 $${BB} \% =\frac{{Walks}}{{Plate}\,{Appearances}}$$

Equation 2: Definition of Walk Rate.

Players who routinely draw walks generally differentiate well between balls and strikes, forcing the pitcher to throw pitches that are easier to hit. Walk rate can also provide information about a hitter’s underlying approach at the plate.

**Strikeout Rate** measures a player’s propensity to strike out. Strikeout rate is defined as

3 $${K}\, \% =\frac{{Strikeouts}}{{Plate}\,{Appearances}}$$

Equation 3: Definition of Strikeout Rate.

Strikeouts are an unequivocally negative outcome for the offense and should be avoided in an at-bat. Although some successful players have high strikeout rates, a high strikeout rate indicates that a batter struggles recognizing pitches or making contact with the ball. A player who strikes out frequently and walks rarely typically has a dim future in baseball.

**Slugging Percentage** measures a player’s propensity to hit for power. Slugging percentage is defined as

4 $${SLG}=\frac{{Total}\,{Bases}}{{At}\,{Bats}}$$

Equation 4: Definition of Slugging Percentage.

Slugging percentage makes use of the fact that not all hits are equally valuable. Although it is an imperfect metric (e.g. doubles are not worth twice as much as singles), it does a decent job of quantifying batting power. Sensorimotor abilities may have different effects on a batter’s ability to hit for contact and ability to hit for power.

Fielder-Independent Pitching measures a pitcher’s run prevention, independent of the ability of the defense behind him. FIP is defined in terms of only variables that cannot be affected by the ability of the defense behind the pitcher.

5 $${FIP}=\frac{13\ast {Home}\,{Runs}+3\ast ({Walks}+{Hit}\,{By}\,{Pitch})-2\ast {Strikeouts}}{{Innings}\,{Pitched}}+{FIP}\,{Constant}$$

Equation 5: Definition of Fielder-Independent Pitching.

According to FanGraphs^27^, “Fielder Independent Pitching (FIP) measures what a player’s ERA would look like over a given period of time if the pitcher were to have experienced league average results on balls in play and league average timing”. It is generally more stable than ERA, since it is a measurement that cancels out the effects of defense and luck. Sensorimotor abilities may be related to pitcher performance, and FIP represents one of the best metrics for quantifying pitcher performance in a game setting.

### Sample Characteristics

Although data was obtained for 308 professional baseball players (149 batters, 159 pitchers), we only examine data for the players with more than 30 at-bats or more than 30 innings pitched to mitigate the statistical noise associated with low sample sizes. This yields a final analyzed data set of 252 players (141 batters, 111 pitchers). Table 1 reports the distribution of age and positional category in this sample. In general, most of the players in the sample are young prospects between 20–25 years old. However, we do have older players in the sample who disproportionately play in the Major Leagues.

**Table 1:** Sample characteristics.

	Batters	Pitchers
**Age**		
Mean (SD)	22.7 (3.9)	23.7 (3.6)
Min–Max	17–37	18–39
**Position**		
#Catchers	19	
#Infielders	65	
#Outfielders	57	

Not all professional leagues are equal. The level of competition in Major League baseball significantly outclasses that of AA baseball, for example. Players in our sample played in leagues ranging from Rookie League to Major League Baseball, which makes player comparison more challenging. Table 2 displays the number of players in our sample who play in each league. Note that some players play in more than one league.

**Table 2:** Distribution of Leagues by Player Type.

	Rookie	A	Adv. A	AA	AAA	Majors
Batters	63	18	33	17	23	14
Pitchers	29	17	24	21	17	13

### Statistical Models

We fit separate models for the five response variables. The models use a common set of predictors. For any model, all parameters are estimated using only the data for that model. For convenience, we use a common notation across models when describing the models, so α_j_ in the OBP model will be distinct from α_j_ in the SLG model, for example.

#### Binomial Response

Since OBP, BB%, and K% are defined as the number of successes divided by the number of opportunities, we use a binomial response for these three variables. Without loss of generality, we present the model for OBP. Let *OB*_*ij*_ denote the number of times that player i reached base in league *j* out of *N*_*ij*_ opportunities between 2012 and 2013. We treat each *OB*_*ij*_ as a realization of a random sample, with the player’s true on base percentage equal to *p*_*ij*_. Each *p*_*ij*_ is a function of the degree of difficulty of getting on base in league *j*, as well as the player’s latent, on-base ability parameter *A*_*i*_. Each *A*_*i*_ is a function of variables X_i_ that include the player’s sensorimotor abilities, a set of indicator variables for position, and age. Putting it together, we have the Bayesian multilevel model^28^

6 $${O}{{B}}_{{ij}} \sim {Binomial}\,({{N}}_{{ij}},\,{{p}}_{{ij}})\,\,{logit}\,({{p}}_{{ij}}) \sim {Normal}\,({\alpha }_{{j}}+{\gamma }_{{j}}{{A}}_{{i}},{\tau }^{-1})\,\,{{A}}_{i}={{X}}_{{i}}^{{T}}\beta $$

Equation 6: On-base percentage model.

Here, *α*_*j*_ represents the degree of difficulty in league *j*, and *γ*_*j*_ represents the impact of ability on performance in league *j*. Accounting for league differences in this way enables us to compare, for example, a 0.400 OBP player in AAA ball to a 0.320 OBP player in the MLB. We constrain *γ*_*j*_ to be positive, so that a higher latent ability level corresponds to a higher probability of reaching base. We use *τ* ^−1^ > 0 to allow for additional player heterogeneity when modeling the *p*_*ij*_. We include all of the sensorimotor variables in *X*_*i*_ with the exception of Go/No-Go, since it is highly correlated with the Eye-Hand Coordination task and has limitations as a task^19^. We transform Depth Perception to the log scale as it is highly right-skewed and model the performance effects of age as linear. Diagnostics indicated that modeling age linearly fits the data reasonably well for all outcome models. We note that our findings were robust to both non-linear models for age and a maximum age threshold, mainly because age and sensorimotor tasks have weak associations in our sample (see also^22^). In addition to standardized age, X_i_ includes an indicator for catcher and an indicator for infielder. Hence, interpretations of all position coefficients are with respect to outfielders. Ultimately, we are interested in performing inference on the posterior distribution of β, which represents the impact of sensorimotor abilities on *A*_*i*_. We use non-informative normal-gamma priors on β and τ; see Section 5 of the Supplementary Materials for details.

#### Normal Response

SLG and FIP are long-run statistical averages over the number of at-bats and innings pitched, respectively. We present the model for SLG below; the model for FIP uses the same format. Let *SLG*_*ij*_ be the slugging percentage for player *i* in league *j* in *N*_*ij*_ at-bats for player *i* in league *j*. By the central limit theorem, as *N*_*ij*_ increases, the sampling distribution of *SLG*_*ij*_ approaches a normal distribution with some mean *μ*_*ij*_ and variance *σ* ^2^/*N*_*ij*_. Because we only included observations with *N*_*ij*_ > 30, the assumption of normality is plausible. We then specify a Bayesian multilevel model conditional on the slugging percentage ability parameters and league adjustment parameters. We have

7 $${SL}{{G}}_{{ij}} \sim {Normal}\,({\mu }_{{ij}},\frac{{\sigma }^{2}}{{{N}}_{{ij}}})\,\,{\mu }_{{ij}} \sim {Normal}\,({\alpha }_{{j}}+{\gamma }_{{j}}{{A}}_{{i}},{\tau }^{-1})\,\,{{A}}_{{i}}={{X}}_{{i}}^{{T}}\beta $$

Equation 7: Slugging percentage model.

The procedure for estimating this model is analogous to the binomial response case, but with an inverse-gamma prior distribution for *σ* ^2^. The prior specifications are provided in Section 5 of the Supplementary Material.

### Model Estimation

The models outlined in Eq. 6 and Eq. 7 are not identifiable since *α*_*j*_, *γ*_*j*_, and A_i_ are unknown and depend upon each other. We overcome this problem by imposing highly concentrated priors on α_j_ and γ_j_, obtained by modeling the game statistics of all professional baseball players between 2012 and 2013 who played in multiple leagues. Details about this process are available in Supplementary Material 4.

The prior means of the league effect parameters *α*_*j*_ and *γ*_*j*_ obtained via the model of game statistics with all professional players are summarized in Tables 3 and 4. In particular, Table 3 illustrates that there are two significant jumps in difficulty in professional baseball. There is a sizable increase in the quality of competition between Rookie baseball and non-rookie minor league baseball (A-AAA). In addition, there is an immense gap between AAA and the Major Leagues. Our model was unable to differentiate significantly between the non-rookie minor leagues. From Table 4, the impacts of ability are consistent across leagues, with the exception of the Major Leagues. With some statistics, such as OBP, latent ability matters less in the Major Leagues than it does in others. With others, such as FIP, it matters much more.

**Table 3:** Posterior Means for *α*_*j*_ displaying the inverse-logit of the means for OBP, BB%, and K% for interpretability. In context, we project that an average professional player will obtain a 0.358 OBP in Rookie ball and a 0.292 OBP in the MLB.

Attribute	Rookie	A	Adv. A	AA	AAA	MLB
logit^−1^ (OBP)	0.358	0.327	0.327	0.322	0.329	0.292
logit^−1^ (BB%)	0.108	0.093	0.096	0.093	0.089	0.071
logit^−1^ (K%)	0.170	0.188	0.184	0.192	0.195	0.232
SLG	0.432	0.384	0.379	0.376	0.397	0.351
FIP	3.013	3.517	3.377	3.613	3.782	4.279

**Table 4:** Posterior Means for *γ*_*j*_. Higher values indicated higher relative impact of ability on the corresponding game statistic, given the model. These values should not be compared across statistics, since they are on different scales.

Attribute	Rookie	A	Adv. A	AA	AAA	MLB
OBP	0.110	0.118	0.109	0.101	0.103	0.060
BB%	0.316	0.304	0.300	0.320	0.305	0.275
K%	0.327	0.356	0.335	0.346	0.345	0.341
SLG	0.059	0.050	0.045	0.041	0.047	0.027
FIP	0.356	0.405	0.383	0.343	0.442	0.556

Once strong prior information on *α*_*j*_ and *γ*_*j*_ is obtained, we estimate the models detailed in Equations 6 and 7, restricting our attention to the seasons of 141 batters and 111 pitchers in our sample with greater than 30 at-bats or innings pitched in each league. While it is reasonable to include data from all 149 batters when estimating the binomial response models, we elect to use the same player pool in all our models for consistency. To facilitate efficient Gibbs sampling and generate comparable coefficients, we standardize all variables in X_i_ with the exception of the position dummy variables. Although measurements of Depth Perception are missing for four batters and four pitchers, the missing values are sampled as part of the Gibbs sampler used to estimate the model^29^ with an independent standard normal prior placed on each of the missing values. We ran the model for three chains of 10,000 iterations after a 1000 iteration burn-in period, and validated it using Markov Chain Monte Carlo diagnostics and posterior predictive checks.

## Results

To start off the analysis, we check to see if performance on the battery of sensorimotor tasks predicts on-field performance. In doing so, for each response variable, we fit two separate models: one with the sensorimotor tasks included and one with only age and position included as control variables. If sensorimotor abilities predict on-field performance, the full model should significantly outperform the reduced model. Following upon this, we report the individual coefficients for each of the models in which sensorimotor abilities added predictive power beyond the control variables.

### WAIC

The Watanabe-Akaike Information Criterion (WAIC) is a useful way to compare two different Bayesian models of a particular response. It uses the log-posterior predictive density as the primary measure of accuracy, with a correction based upon the effective number of parameters in the model^30^. Asymptotically, it can be shown that WAIC approaches the results obtained via leave-one-out cross-validation^31^. For each of the five models, we use WAIC to compare the full model with the sensorimotor task results included in the design matrix to the reduced model that only accounts for position and age. If sensorimotor variables add predictive power above and beyond that of the control variables, then the WAIC of the full model should be lower than that of the reduced model.

Table 5 compares the WAIC of the full model to that of the reduced model for each of the five response variables. As indicated by the lower values for the Full, relative to the Reduced model, performance on the Nike Sensory Station tasks is predictive of OBP, BB%, and K%. However, sensorimotor abilities do not predict either SLG or FIP. We therefore present coefficient summaries for OBP, BB%, and K% in the sections below. Summaries for SLG and FIP can be found in the supplementary materials.

**Table 5:** WAIC Model Comparison. Lower values for the full models relative to the reduced OBP, BB%, and K% models indicate that the added variables in the full models add meaningful predictive power.

	OBP	BB%	K%	SLG	FIP
Full Model	1210.8	1075.8	1276.4	403.4	363.8
Reduced Model	1226.4	1084.4	1284.6	403.1	361.9

### On-Base Percentage

**Figure 1:**
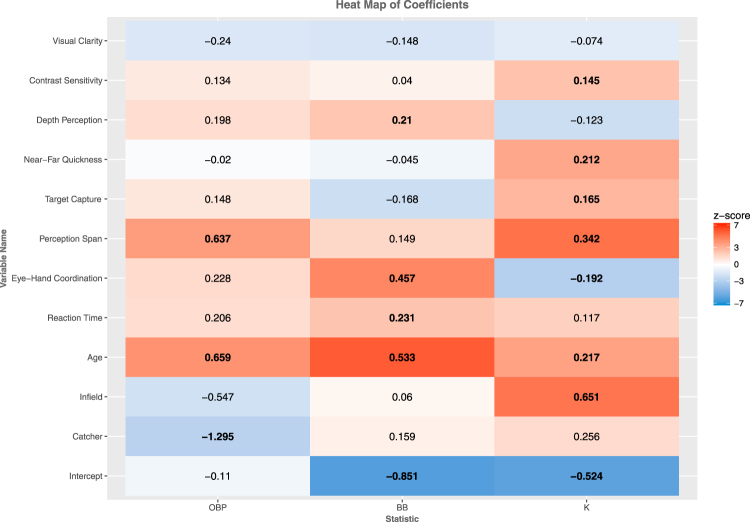
Heat map of β Coefficients. The darker the color, the closer the posterior tail probability gets to zero (indicating evidence of an association). Values for which the 95% credible interval excludes zero are bolded.

The posterior means, standard deviations, and 95% credible intervals for the coefficients β are presented in Table 6 for the full OBP, BB%, K% models, and in Supplementary Material 6 for SLG and FIP. The control covariates that are included in both the full and reduced models are indicated in the left sidebar. Variables for which 0 falls outside the 95% credible intervals are bolded. In general, bolded positive coefficients indicate that there is greater than 95% probability that the sensorimotor ability measured in the task has an association with on-field performance. To illustrate the posterior tail probabilities, a heat map of the z-scored coefficients for OBP, BB% and K% is given in Fig. 1.

**Table 6:** Mean coefficients, standard deviations, and 95% credible intervals for each model variable are shown for (A) on-base percentage, (B) walk rate, and (C) strikeout rate. Values for which the 95% credible interval excludes zero are bolded.

		On-Base Percentages				(B) Walk Rate				(C) Strikeout Rate			
		Mean	SD	2.5%	97.5%	Mean	SD	2.5%	97.5%	Mean	SD	2.5%	97.5%
Only Full Model	Visual Clarity	−0.24	0.17	−0.59	0.10	−0.15	0.10	−0.35	0.05	−0.08	0.07	−0.21	0.06
	Contrast Sensitivity	0.13	0.16	−0.18	0.45	0.04	0.09	−0.14	0.23	**0**.**14**	**0**.**06**	**0**.**02**	**0**.**26**
	Depth Perception	0.19	0.16	−0.12	0.50	**0**.**21**	**0**.**10**	**0**.**02**	**0**.**40**	−0.12	0.07	−0.26	0.02
	Near-Far Quickness	−0.02	0.15	−0.32	0.28	−0.05	0.09	−0.23	0.14	**0**.**21**	**0**.**07**	**0**.**09**	**0**.**34**
	Target Capture	0.15	0.16	−0.16	0.47	−0.16	0.09	−0.35	0.01	**0**.**16**	**0**.**06**	**0**.**04**	**0**.**29**
	Perception Span	**0**.**64**	**0**.**17**	**0**.**31**	**0**.**99**	0.15	0.10	−0.04	0.34	**0**.**34**	**0**.**07**	**0**.**21**	**0**.**47**
	Eye-Hand Coordination	0.22	0.17	−0.11	0.56	**0**.**46**	**0**.**10**	**0**.**26**	**0**.**67**	−**0**.**19**	**0**.**07**	−**0**.**32**	−**0**.**06**
	Reaction Time	0.21	0.17	−0.11	0.55	**0**.**23**	**0**.**11**	**0**.**03**	**0**.**44**	0.12	0.07	−0.02	0.26
Both	Age	**0**.**66**	**0**.**17**	**0**.**34**	**1**.**00**	**0**.**53**	**0**.**09**	**0**.**36**	**0**.**71**	**0**.**22**	**0**.**06**	**0**.**09**	**0**.**34**
	Infield	−0.53	0.31	−1.15	0.08	0.05	0.19	−0.33	0.43	**0**.**65**	**0**.**13**	**0**.**40**	**0**.**91**
	Catcher	−**1**.**28**	**0**.**49**	−**2**.**25**	−**0**.**35**	0.15	0.29	−0.40	0.72	0.26	0.19	−0.12	0.64
	Intercept	−0.13	0.23	−0.57	0.31	−**0**.**84**	**0**.**14**	−**1**.**12**	−**0**.**57**	−**0**.**52**	**0**.**09**	−**0**.**71**	−**0**.**34**

From the OBP model results we observe that performance on the Perception Span task, which measures the ability to remember and recreate visual patterns, is associated with an increased ability to reach base. Moreover, the size of the coefficient is comparable to that of age (SD = 3.8 years), a remarkable result considering it is well known that older players tend to perform better than younger players in professional baseball due to survivorship bias. For interpretation, suppose player X is a 23-year-old outfielder with completely average abilities as a professional baseball player. The model predicts his OBP in the MLB to be 0.292. We expect a similar player who scores one standard deviation higher on the Perception Span task would have an OBP of 0.300, a nontrivial difference. While the coefficients of the other tasks trend positive, there is simply not enough data to draw strong conclusions about them.

### Walk Rate (BB%) and Strikeout Rate (K%)

Although 41% of the variation in OBP is explained by the combination of strikeout rate (K%) and walk rate (BB%; Table 7), the two metrics are nearly completely uncorrelated in our sample, even after removing batters with fewer than 50 plate appearances. This suggests that walk rate and strikeout rate are both components of on-base percentage but capture different features of a batter. As such, the sensorimotor abilities that affect a player’s ability to draw walks differ from those that affect a player’s ability to avoid strikeouts.

**Table 7:** Results of an OLS Regression of OBP on BB% and K%. BB% and K% are both significant predictors of OBP, but BB% is a much better predictor.

	Estimate	Std. Error	T value	Pr(>|t|)
(Intercept)	0.3020	0.0127	23.72	0.0000
BB%	0.7203	0.0902	7.99	0.0000
K%	−0.2077	0.0449	−4.63	0.0000

The results presented in Table 6 bear this out. Superior performance on the tasks that measure a player’s ability to quickly identify and react to visual stimuli, Eye-Hand Coordination and Reaction Time, were found to be associated with an increased ability to draw walks. For example, our model predicts player X to obtain a walk rate of 7.1% in the MLB, but predicts a similar player with a one standard deviation superior score on the hand-eye coordination task to have a walk rate of 8.0%. On the other hand, superior performance on tasks that measure a player’s spatial recognition and memory, such as Near-Far Quickness, Target Capture, and Perception Span, was found to be associated with an increased ability to avoid strikeouts. In context, our model predicts the strikeout rate of player X to be 23.2% in the MLB. A player similar to player X who scores one standard deviation better on the Perception Span task is predicted to have a strikeout rate of 21.2%. It is surprising that Eye-Hand Coordination was found to be significant in the opposite direction than we would expect a priori, which motivates further study.

## Discussion

The specific roles of vision, perception and motor control in interceptive sports such as baseball and cricket have been a hotly debated topic for years^3,9,32,33^. In the current study, we shed new light on this debate by using real-world data collected from a large sports performance program launched by Nike Inc. Through Bayesian hierarchical latent variable modeling of the relationship between psychometric performance on the task battery and season-wise game statistics, we find that sensorimotor abilities predict on-base percentage, walk rate, and strikeout rate, but not slugging percentage or fielder-independent pitching.

The observation that better sensorimotor abilities generally correlate with better on-base percentage, walk rate, and strikeout rate is largely intuitive since it is expected that players draw on these skills to project the location of the pitch through the strike zone and decide whether to swing or not. Conversely, the ability to hit for power, captured by slugging percentage, should have more to do with strength, bat speed, and swing plane than sensorimotor abilities. Pitchers rely on a strong arm, consistent mechanics, and a varied repertoire to prevent runs, attributes that are superficially unrelated to sensorimotor abilities.

Among the individual tasks tested, Perception Span’s relationship with on-field performance produced the strongest relationships, with better scores strongly associated with both increased on-base percentages and reduced strikeout rates. In addition, performance on the Perception Span task exhibits some association with both higher walk rates and increased slugging percentages, though the evidence is not conclusive. This task measures the ability to remember and recreate visual patterns and may reflect visual recognition abilities that have previously been tied to batting performance in small samples of players (N = 20) tested with Tachistoscopic methods^7^ and in a conference paper reporting relationships in collegiate players^8^. For reference, across the tested models we find expected age and position effects with stronger batting performance for outfields and older batters. The fact that the Perception Span effects were within the range of magnitudes for age and position effects indicates the relatively strong contribution that visual recognition abilities may play in batting performance.

A number of other tasks correlate with higher walk rates, including Depth Perception, Eye-Hand Coordination and Reaction Times. The observation that Eye-Hand Coordination and Reaction Time are positively correlated with walk rate indicates that the ability to quickly react to visual stimuli is highly influential in a player’s ability to draw walks. The positive relationship with Depth Perception supports previous findings indicating that binocular vision contributes to precisely projecting the location of a pitched baseball^34^. Furthermore, past research comparing pitchers and hitters on the Sensory Station battery found better performance for professional hitters, relative to pitchers, on both the Visual Clarity and Depth Perception tasks^22^, suggesting that better depth disparity differentiates highly experienced athletes who bat from those who pitch.

The model linking sensorimotor abilities to strikeout rates offers a mixed view of the relative contributions of sensorimotor skills towards avoiding strikes. The pattern of results indicates that better performance on the Perception Span, Near-Far Quickness, Target Capture, and Contrast Sensitivity tasks is associated with an increased ability to avoid strikeouts. However, it is surprising that worse Eye-Hand Coordination is associated with reduced strikeout rates, though it has a relatively weak coefficient.

Overall, the current results suggest performance contributions from aspects of both receptive vision that constitutes visual hardware (Contrast Sensitivity and Depth Perception) and visual software that enables the processing of this information (Target Capture, Near-Far Quickness, Perception Span, Eye-Hand Coordination, Reaction Time). While on balance it appears that tasks measuring visual software are more predictive of on-field performance in professional baseball, it is important to note that these tasks capture a diverse set of physiological and psychomotor constructs, including receptive visual processing, oculomotor control, eye-limb coordination and visual decision making. Nonetheless, the current findings provide novel evidence of the importance of these abilities towards on-field achievement.

In light of the current findings, it is worth noting several strengths and weaknesses in the approach. First, this dataset reflects one of the largest samples of high-level baseball players tested on a consistent battery of psychometric tasks. These tasks were presented with video instructions and conducted by trained and certified administrators, providing some assurance towards data quality, but also opening the possibility that differences between the testing environment (athletic training facilities) and active game situations may have contributed to unexpected relationships such as the counter-intuitive observation that lower eye hand coordination scores correlated with higher strikeout rates. Further, the latent approach to modeling league heterogeneity offers a systematic means by which to incorporate data from multiple leagues, while also scaling production in each league to accurately reflect the relative difficulty of that league in that year. Nonetheless, it is important to note that while the individual tasks in the battery have been identified as important abilities for sports performance^17^ the choice to include multiple measures in the battery comes with a tradeoff of fewer trials (and less sensitivity) for each measure. As such, it will be important to replicate these findings with tasks that each are strongly powered to characterize the individual sensorimotor abilities under consideration.

### Future Work

One interpretation of the strong relationship between Perception Span and batting performance is that the ability to store pitches in spatial working memory, and subsequently recognize them, helps batters avoid strikeouts and reach base more frequently. There may be evidence for this empirically, since pitchers obtain the highest strikeout rates and allow the lowest on-base percentage when seeing batters for the first time. Each time a batter faces a pitcher, his on-base percentage improves and strikeout rate declines^35^, in part because he has “seen” the pitcher’s repertoire before and filed it away into memory, making for easier recollection and recognition during subsequent meetings. Future research may examine whether players with high scores on the Perception Span task perform better against pitchers during the second and third times through the order, above and beyond the improvement expected of them. To do this, at-bat level data (such as that available through PITCHf/x) will be needed, rather than aggregated season statistics.

If the present and future results speak to underlying building blocks of baseball expertise, how can they be used to improve baseball performance? This question lies at the heart of efforts to implement “sports vision training” programs^36,37^, based on the notion that practice with demanding visual, perceptual, cognitive, or oculomotor tasks can improve the ability to process and respond to what is seen, thereby improving athlete performance. The literature has examined training techniques that target anticipation and decision-making abilities of athletes^38^, as well as new digital technologies that train general visual, perceptual and cognitive skills critical for sporting performance^25,39,40^. Ultimately, the ability to determine which visual and motor characteristics are related to performance will focus research on specific training programs, enabling athletes to make the most of their system physiologies. The current findings are a step in this direction.

## Electronic supplementary material

Supplementary Material
