# Supplementary material for: Sensorimotor abilities predict on-field performance in professional baseball
Source: Sci Rep. 2018 Jan 8;8:116. doi: 10.1038/s41598-017-18565-7 (PMC5758703; doi:10.1038/s41598-017-18565-7)
Supplement: Supplementary file 1 — Supplementary Material [file 41598_2017_18565_MOESM1_ESM.doc]

**Supplementary Materials**

**Sensorimotor abilities predict on-field performance in professional baseball**

Kyle Burris^a^

Kelly Vittetoe^b^

Ben Ramger^b^

Sunith Suresh^a^

Surya Tokdar^a^

Jerome Reiter^a^

L. Gregory Appelbaum^b^

^a^ Department of Statistical Science, Duke University, Durham, NC, 27708, USA

^b^ Department of Psychiatry and Behavioral Science, Duke University School of Medicine, Durham, NC,

**Correspondence**

Dr. Greg Appelbaum

Duke University Hospital

400 Trent Dr.

Durham NC, 27710

Phone: 919-613-7664. Fax: 919-681-8744

E-mail: [greg@duke.edu](mailto:greg@duke.edu)

**Supplementary Material 1: Sensorimotor Assessments**

The Sensory Stations consist of a battery of nine computerized sensorimotor tasks (illustrated below), each designed to evaluate a specific facet of a participant’s visual-motor abilities. The first five tasks were completed using a handheld Apple iPod Touch, standing 4.9 m from the station. The last four tasks were completed at arm’s length from the touchscreen monitor. Four of the tasks – Visual Clarity, Contrast Sensitivity, Depth Perception, and Target Capture – operated on staircase schedules in which subsequent stimulus difficulty increased following a correct response and decreased following an incorrect response. For these tasks, scores were calculated as the final step according to response accuracy on the staircase schedule. All tasks were preceded by video instructions. Procedures and descriptions for each task are provided below, and detailed descriptions can be found in Erickson et al., (2011) and Wang et al., (2015).

The **Visual Clarity** task measures visual acuity for fine details at a distance using a black Landolt ring – an incomplete ring with a small gap oriented in one of the four cardinal directions. Participants were asked to swipe on the iPod in the direction that corresponded to the orientation of the gap in the ring. The task was completed in three separate rounds: one with an occluder covering the right eye, then the left, then a final round with both eyes uncovered. Visual Clarity scores were taken as the average of these three conditions.

The **Contrast Sensitivity** task measures the minimum resolvable difference in contrast at a distance. Participants were presented with four black rings on a light gray background and asked to indicate which ring contained a pattern of dark gray concentric circles by swiping on the iPod in the direction corresponding to the patterned ring.

The **Depth Perception** task measures how quickly and accurately participants are able to detect differences in depth at a distance using liquid crystal glasses. Here, four black rings were presented and participants were asked to swipe in the direction of the ring that appeared to have depth. The task was completed three times: once facing towards the screen, once facing to the left and looking over the right shoulder, and once facing right and looking over the left shoulder. Depth Perception scores were taken as the average of these three conditions.

The **Near-Far Quickness** task measures the number of near and far targets that can be correctly reported in 30 seconds. Participants aligned the top of the iPod with the bottom edge of the large monitor then swiped in the direction of the gap in the Landolt ring that appeared on either the iPod or larger monitor screen. Participants were instructed to respond as quickly as possible, and the ring only moved from one screen to another following a correct response. Participants continued to respond until they answered correctly or ran out of time. Near-Far Quickness scores were the total number of correct responses made in 30-seconds.

The **Target Capture** task measures the speed at which participants can shift attention and recognize peripheral targets. A small black Landolt ring was briefly presented in one of the four corners of the monitor, and participants were asked to swipe on the iPod in the direction corresponding to the gap in the ring. Following a correct answer, the ring was presented for a shorter duration, and for a longer duration following an incorrect answer, per the staircase procedure. Because this task was performed on a duration staircase, the final accuracy step reflected the minimum stimulus duration according to accuracy on the staircase schedule.

The **Perception Span** task measures the capacity of spatial working memory. As participants stood at arm’s length from the monitor, a grid of empty black circles was presented, and a subset was filled briefly with green dots that disappeared after 100 milliseconds. Participants were asked to recreate the pattern on each trial by touching the circles that had previously contained the green dots. There were eleven total possible pseudo-randomized trials with increased grid sizes and increasing number of green dots presented at each level. Perception Span scores were computed as the total number of correctly identified dots minus the number of missed or falsely identified dots across all of the trials.

The **Eye Hand Coordination** task measures the speed at which participants can make visually-guided hand responses to rapidly changing targets. A grid of 48 evenly spaced black rings was presented on the screen. When a green dot appeared in one of the rings, participants touched the dot as quickly as possible. The dot then relocated to another ring for a total succession of 96 dots. The score for Eye Hand Coordination was the total time it took to complete the sequence.

The **Go/No-Go** task measures the ability to execute and inhibit visually guided hand responses in the presence of “go” and “no-go” stimuli. Similar to the previous task, a grid of 48 rings was presented; however, in this task the dots could appear either green or red. Participants tried to touch the green dots as quickly as possible while avoiding red dots. 96 dots were presented for 500 milliseconds each before disappearing, and the total score was calculated as the number of green dots touched minus the number of red dots touched.

The **Response Time** task measures how quickly participants react and respond to a simple visual stimulus. Two rings were shown on each side of the large monitor. Participants began with their dominant hand in the “starting” ring, while their body was oriented in front of the “landing” ring on the opposite side of the screen. When the landing ring turned green, participants moved their hand from the starting ring to the landing ring as quickly and accurately as possible. A total of seven separate trials were completed, and participants had the opportunity to repeat up to two of these trials if any were slower than two standard deviations from the mean. Response Time scores were taken as the average of the seven best trials.


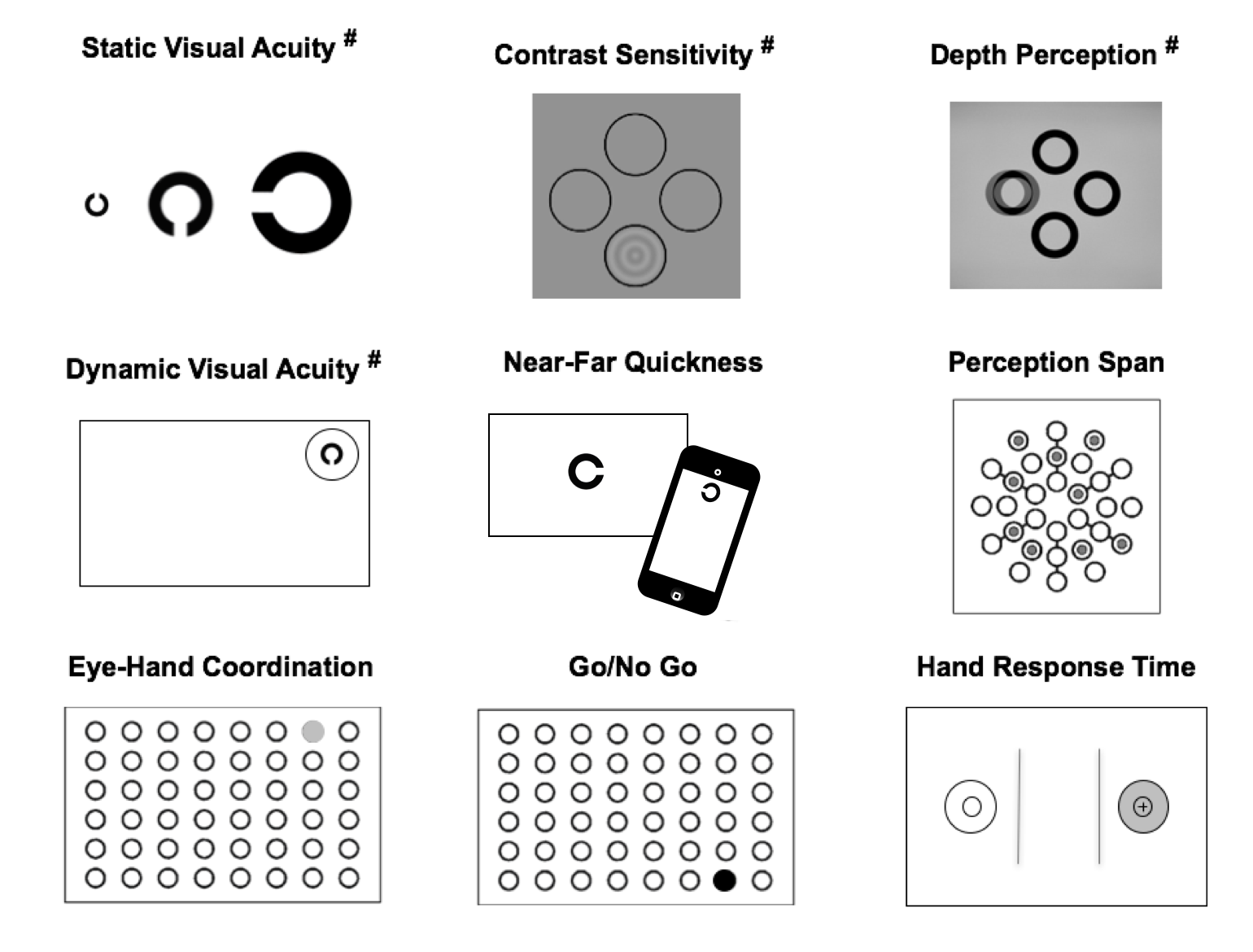


Illustrations of the nine perceptual and visual-motor tasks included in the Nike SPARQ Sensory Station battery. # indicates tasks that performed under a staircase schedule.

**Supplementary Material 2: Performance Distributions for the Sensory Station tasks.**


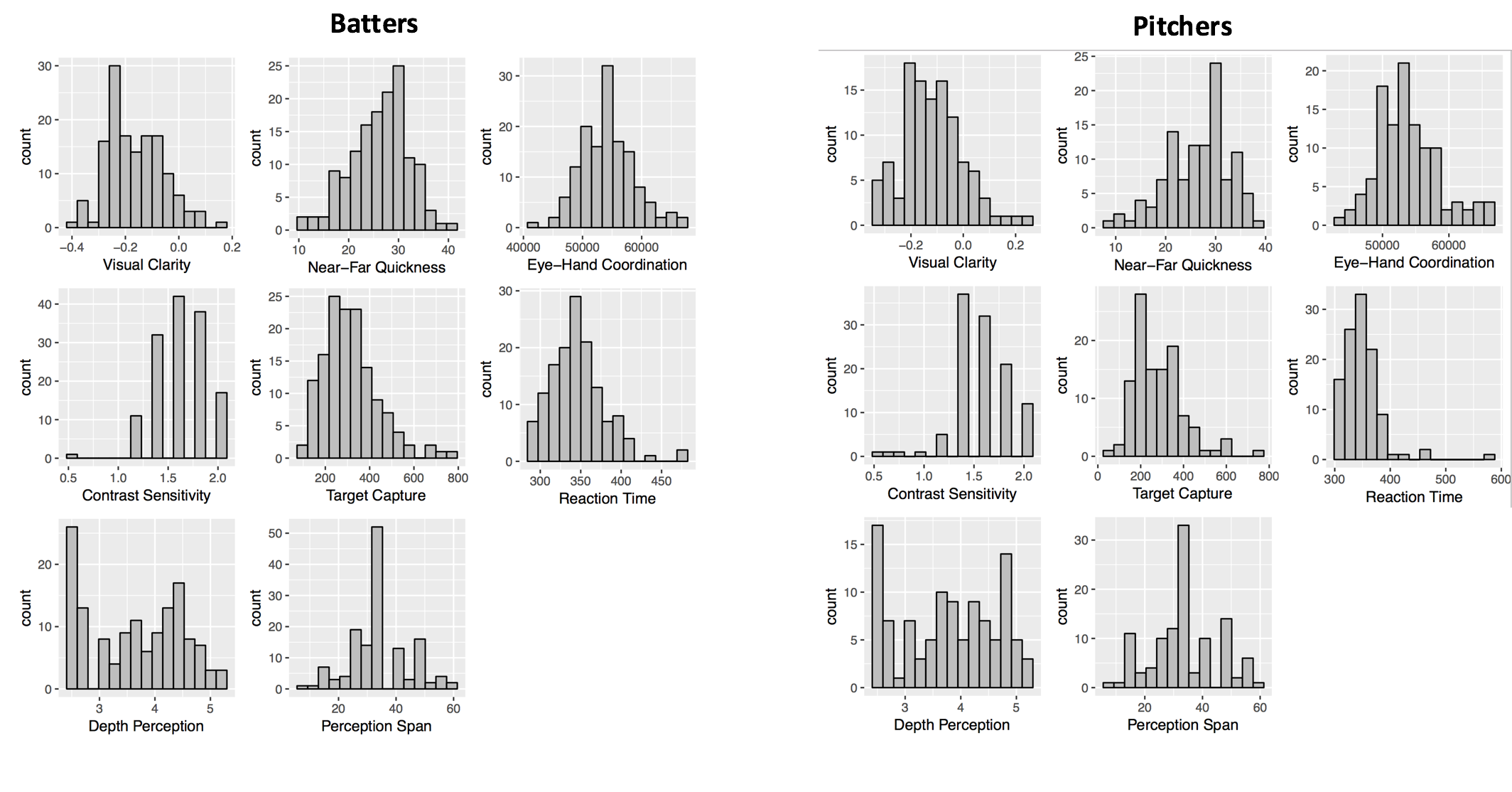


*Histograms of behavioral performance on the Sensory Station tasks for (A) batters and (B) pitchers.*

**Supplementary Material 3**


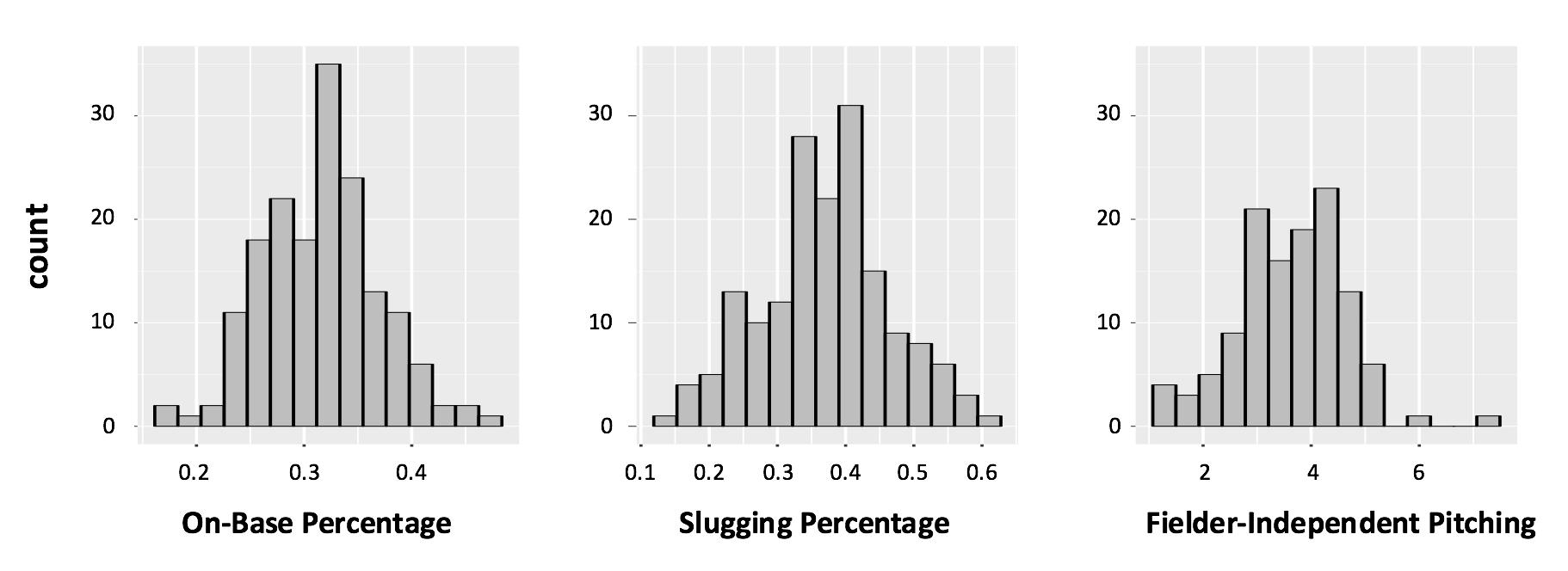


*Histograms of game performance statistics*

**Supplementary Material 4: League Effect Models**

To do estimate α_j_ and γ_j_, we first scrape publicly available minor and major league play-by-play data between 2012 and 2013 from MLB.com using the pitchRx package in the R language (Sievert, 2015). We collate the statistics of all players (1695 batters, 1053 pitchers) who played in multiple leagues during that period. By examining the difference in player performance between the leagues, we quantify the degree of difficulty of each league with a separate Bayesian model. For example, if the Major League is more difficult than AAA, we should expect a player who plays in both leagues to register a lower on-base percentage in the Major League than in AAA.

For each of the five game statistic variables, we estimate the corresponding model detailed in Eqs. 1 and 2, using the data of all players who played in multiple leagues between 2012 and 2013. Since we do not have sensorimotor measurements for these players, we instead place a standard normal prior on each A_i_. Because we assume that increased ability corresponds to improved performance for each game statistic, we impose a half-Cauchy prior on γ_j_, as well as conjugate Normal/Gamma priors for α_j_ and τ.

For the initial on-base percentage model, we use the following specification and priors.

| 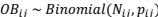 | 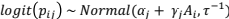 | 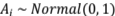 |
| --- | --- | --- |
| 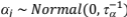 | 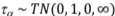 |  |
| 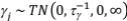 | 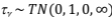 | 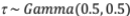. |

where TN(μ, σ, a, b) is the normal distribution truncated to a lower bound a and upper bound b. The initial models for BB% and K% models have similar specifications, with the exception that γ_j_ in the K% model is bounded above by zero, since players with higher ability strike out less frequently.

For the initial slugging percentage model, we use the following specification and priors.

| 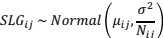 | 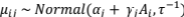 | 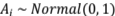 |
| --- | --- | --- |
| 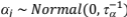 | 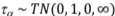 | 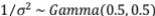 |
| 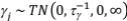 | 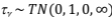 | 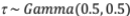. |

The specification for the FIP model is the same as that for the SLG model, with the exception that γ_j_ in the FIP model is bounded above by zero, since players with higher ability record lower values for FIP.

For each model, we draw thousands samples of the parameters from the joint posterior distribution via Gibbs sampling. We compute the posterior means and variances for all parameters, including α_j_ and γ_j_ . These means and variances are used in the prior distributions for the final models, as described **in Section 5 of the Supplementary Material.**

**Supplementary Material 5: Specifications for Final Models**

As mentioned in the main body of the article and in Section 4 of the Supplementary Material, we first fit an initial model to obtain concentrated priors for α_j_, γ_j_, τ, and σ^2^. We use the posterior means and variances to form prior distributions in the final models used to assess the relationship between sensorimotor abilities and on-field performance. For any initial model, let the
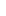
 and
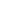
 be the posterior mean and standard deviation of τ, respectively, computed as described in Section 4 of the Supplementary Material. We use analogous notation for the posterior means and standard deviations for the other parameters. We emphasize that each outcome variable has its own set of parameters, although we use a common notation for convenience.

The final on-base percentage model used to produce the results in **Table 6**, including all prior distributions, is as follows:

| 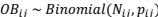 | 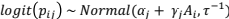 | 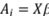 |
| --- | --- | --- |
| 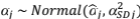 | 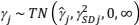 | 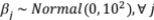 |
|  | 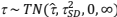 | 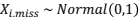 |

The specifications for the BB% and K% models are identical, with the exception that γ_j_ in the K% model is bounded above by zero, since players with higher ability strike out less frequently.

The final slugging percentage model used to produce the results in Table 6, including all prior distributions, is as follows.

| 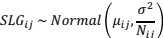 | 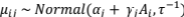 | 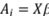 |
| --- | --- | --- |
| 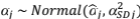 | 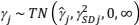 | 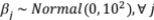 |
| 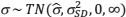 | 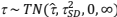 | 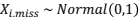 |

The specification for the FIP model is the same as that for the SLG model, with the exception that γ_j_ in the FIP model is bounded above by zero, since players with higher ability record lower values for FIP.

**Supplementary Material 6: Posterior Distributions**

|  |  | 1. Slugging Percentage | | | | (B) Pitcher Independent Fielding | | | |
| --- | --- | --- | --- | --- | --- | --- | --- | --- | --- |
|  |  | Mean | SD | 2.5% | 97.5% | Mean | SD | 2.5% | 97.5% |
| Full Model | Visual Clarity | -0.01 | 0.15 | -0.30 | 0.28 | -0.24 | 0.25 | -0.71 | 0.26 |
|  | Contrast Sensitivity | -0.14 | 0.14 | -0.41 | 0.15 | 0.07 | 0.24 | -0.40 | 0.54 |
|  | Depth Perception | 0.18 | 0.14 | -0.08 | 0.45 | **-0.68** | **0.22** | **-1.12** | **-0.26** |
|  | Near-Far Quickness | 0.00 | 0.14 | -0.26 | 0.27 | 0.25 | 0.23 | -0.20 | 0.70 |
|  | Target Capture | 0.15 | 0.14 | -0.12 | 0.43 | -0.11 | 0.21 | -0.52 | 0.29 |
|  | Perception Span | **0.29** | **0.14** | **0.01** | **0.58** | 0.21 | 0.23 | -0.25 | 0.66 |
|  | Eye-Hand Coordination | 0.05 | 0.15 | -0.25 | 0.34 | -0.18 | 0.20 | -0.58 | 0.20 |
|  | Reaction Time | 0.09 | 0.15 | -0.21 | 0.40 | -0.07 | 0.20 | -0.47 | 0.34 |
| Reduced | Age | **0.44** | **0.15** | **0.15** | **0.72** | **0.55** | **0.21** | **0.15** | **0.96** |
|  | Infield | **-0.60** | **0.28** | **-1.13** | **-0.05** |  |  |  |  |
|  | Catcher | **-1.40** | **0.41** | **-2.21** | **-0.60** |  |  |  |  |
|  | Intercept | 0.07 | 0.20 | -0.33 | 0.45 | -0.32 | 0.21 | -0.74 | 0.07 |

*Mean coefficients, standard deviations, and 95% credible intervals for each model variable are shown for (A) slugging percentage and (B) Pitcher Independent Fielding. Values for which the 95% credible interval excludes zero are bolded.*

**Supplemental References**

Erickson, G. B., Citek, K., Cove, M., Wilczek, J., Linster, C., Bjarnason, B., & Langemo, N. (2011). Reliability of a computer-based system for measuring visual performance skills. *Optometry, 82*(9), 528-542. doi:10.1016/j.optm.2011.01.012

Sievert, C. (2015). pitchRx: Tools for Harnessing 'MLBAM' 'Gameday' Data and Visualizing 'pitchfx' (Version R package version 1.8.2.).

Wang, L., Krasich, K., Bel-Bahar, T., Hughes, L., Mitroff, S. R., & Appelbaum, L. G. (2015). Mapping the structure of perceptual and visual-motor abilities in healthy young adults. *Acta Psychol (Amst), 157*, 74-84. doi:10.1016/j.actpsy.2015.02.005
